# Induction of c-Cbl contributes to anti-cancer effects of HDAC inhibitor in lung cancer

**DOI:** 10.18632/oncotarget.3489

**Published:** 2015-03-08

**Authors:** Tzu-Tang Wei, Yu-Chin Lin, Pei-Hua Lin, Jin-Yuan Shih, Chia-Wei Chou, Wei-Jan Huang, Yu-Chih Yang, Pei-Wen Hsiao, Ching-Chow Chen

**Affiliations:** ^1^ Department of Pharmacology, College of Medicine, National Taiwan University, Taipei, Taiwan; ^2^ Department of Internal Medicine, Far-Eastern Memorial Hospital, New Taipei, Taiwan; ^3^ Department of Internal Medicine, National Taiwan University Hospital, Taipei, Taiwan; ^4^ Graduate Institute of Pharmacognosy, Taipei Medical University, Taipei, Taiwan; ^5^ Agricultural Biotechnology Research Center, Academia Sinica, Taipei, Taiwan

**Keywords:** c-Cbl, EGFR, HDAC inhibitors, lung cancer

## Abstract

Here we found loss of c-Cbl, an E3 ligase, expression in non-small cell lung cancer (NSCLC) compared with its adjacent normal tissue in patient specimens. HDAC inhibition by WJ or knockdown of HDAC 1, HDAC2, HDAC3 or HDAC6 all induced c-Cbl. Ectopic expression of c-Cbl induced decreased EGFR, inhibited growth in NSCLC cells. Knockdown of EGFR inhibited NSCLC growth. Mutation of EGFR at Y1045 decreased WJ-induced growth inhibition as well as *in vivo* anti-cancer effect and EGFR degradation mediated by WJ. Time-lapse confocal analysis showed co-localization of c-Cbl and EGFR after WJ treatment. Furthermore, WJ inhibited lung tumor growth through c-Cbl induction in orthotopic and tail vein injected models. C-Cbl up-regulation induced by HDACi is a potential strategy for NSCLC treatment.

## INTRODUCTION

Casitas B-lineage lymphoma (c-Cbl) protein is an E3 ubiquitin ligase regulating intracellular signaling [1]. Its dysfunction mutation has been reported to induce myeloid neoplasm while overexpression inhibits tumor growth in xenograft models [2, 3], implicating its tumor suppressive role. C-Cbl acts through E3 ubiquitin ligase activity to promote epidermal growth factor receptor (EGFR) internalization and lysosome degradation [1, 4]. Over-expression of EGFR is frequently found in epithelial cancers and correlated with clinical aggressiveness and poor outcome [5, 6], suggesting that targeting EGFR is an important strategy for cancer treatment. Thus, up-regulation of c-Cbl might be effective. Clinically, anti-EGFR monoclonal antibody and EGFR tyrosine kinase inhibitor (TKI) have already led to great success in head and neck, colorectal, and non-small cell lung cancers [7-9].

Non-small cell lung cancer (NSCLC) is a worldwide cancer usually diagnosed at advanced stage with poor outcome [10]. Platinum-based doublet chemotherapy remains the mainstay for advanced NSCLC, but toxicities including leukopenia, nephrotoxicity, or neurotoxicity hinder its application [11-13]. Although EGFR tyrosine kinase inhibitor leads to a great treatment advance of NSCLC, only a subgroup with EGFR activating mutation benefit from it.

Transcription factor-mediated gene expression is regulated by histone modification, in which histone acetylation induces chromatin relaxation to facilitate this event [14]. The extent of histone acetylation is controlled by the balance between histone deacetylases (HDACs) and histone acetyltransferases (HATs) [14]. Overexpression of HDACs associated with transcription repression inactivates tumor suppressor genes, leading to carcinogenesis and tumor progression [15]. Therefore, HDACs are therapeutic targets for cancer treatment, and HDAC inhibitors have been reported to exert clinical efficacy against hematological malignancies and preclinical activity for solid tumors [16]. Its mechanisms include p21 induction for growth arrest, apoptosis, autophagic cell death, mitotic failure, senescence, anti-angiogenesis by HIF-1 down-regulation, induction of reactive oxygen species (ROS), and inhibition of heat shock protein 90 (HSP90) [17]. In this study, we found c-Cbl was lost in NSCLC patients and disclosed a mechanism that HDAC inhibition could induce c-Cbl up-regulation, in which histone lysine acetylation and transcription factor SP1 play important roles. We synthesized an hydroxamate-based HDAC inhibitor, WJ, which was more potent than SAHA to inhibit HDAC and tumor growth. WJ induced c-Cbl up-regulation to degrade EGFR through lysosome pathway, and knockdown of c-Cbl reversed WJ-induced anti-cancer effect. WJ inhibited-lung tumor growths in orthotopic and tail vein injected mouse models were abolished by Y1045 EGFR mutation, indicating the critical role of EGFR in the anti-cancer effect of HDAC inhibition. Therefore, c-Cbl induction by HDAC inhibition is a promising strategy to treat lung cancer. This finding contributes to the anticancer mechanism of HDAC inhibitors in lung cancers.

## RESULTS

### Tumor suppressive role of c-Cbl in lung cancer and effect of HDAC inhibitor on c-Cbl induction

**Figure 1:**
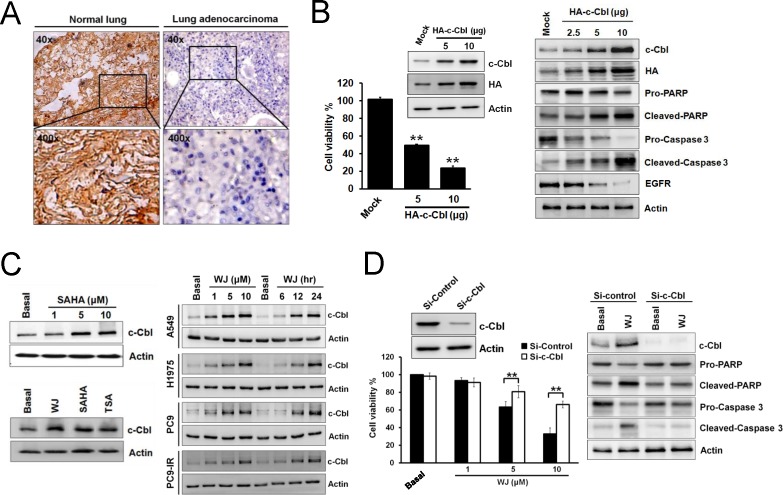
Induction of c-Cbl inhibited cell proliferation and promoted apoptosis in lung adenocarcinoma cells A, IHC staining of c-Cbl expression in normal bronchi and lung adenocarcinoma tissues from patients with lung adenocarcinoma. B, Effects of overexpressed c-Cbl on cell viability after 48 hours in A549 cells (left panel). Dose-dependent effects of overexpressed c-Cbl on PARP and pro-caspase 3 cleavages and EGFR expression after 48 hours in A549 cells (right panel). C, Effect of WJ, SAHA or TSA on c-Cbl expression in A549 cells. A549 cells were treated with 1, 5, 10 μM SAHA for 24 hours (left upper panel). In addition, cells were treated with 5 μM WJ, 5 μM SAHA or 1 μM TSA for 24 hours (left lower panel). NSCLC cells were treated with 1, 5, 10 μM WJ for 24 hours, or treated with 5 μM WJ for 6, 12, 24 hours (right panel). Total cell lysates were prepared and western blot was performed using indicated antibiodies. D, Knockdown of c-Cbl reversed WJ-mediated growth inhibition after WJ treatment for 48 hours in A549 cells (left panel). The cell viability was measured by MTT assay. ^**^P<0.01 versus basal. Effect of siRNA-mediated knockdown of c-Cbl on WJ-induced EGFR degradation and PARP and pro-caspase 3 cleavages in A549 cells after 48 hours (right panel).

Since c-Cbl may play a tumor suppressive role, its expression in 11 lung adenocarcinoma specimens from patients was evaluated by immunohistochemical (IHC) staining. Clinical characteristics of patients are summarized (Supplementary Table S1). Loss of c-Cbl expression was found in cancer part compared to normal part of tissues (Figure 1A). The effect of c-Cbl on cell viability was evaluated by overexpression of c-Cbl into A549 cells. c-Cbl overexpression inhibited cell proliferation and induced PARP and pro-caspase 3 cleavages (Figure 1B). It also down-regulated EGFR expression (Figure 1B), an important oncoprotein in lung cancer. Since c-Cbl is a tumor suppressor in lung adenocarcinoma, we screened a series of small molecules and found that HDAC inhibitor (HDACi) SAHA could induce c-Cbl expression (Supplementary Figure S1A and Figure 1C). Therefore, an HDACi, WJ, which was more potent than SAHA was employed [18]. WJ induced c-Cbl expression in NSCLC cells in a dose- and time-dependent manner (Supplementary Figure S1B and Figure 1C). It showed greater growth inhibitory effect on various NSCLC cells and less toxicity on normal fibroblasts (MEF and HS68) compared to SAHA (Supplementary Table S2). WJ-induced growth inhibition and apoptosis were reversed by the knockdown of c-Cbl (Figure 1D), indicating that c-Cbl played a role in HDACi-induced anti-cancer effect.

### A HDAC inhibitor induced c-Cbl to attenuate EGFR expression and its downstream signaling

**Figure 2:**
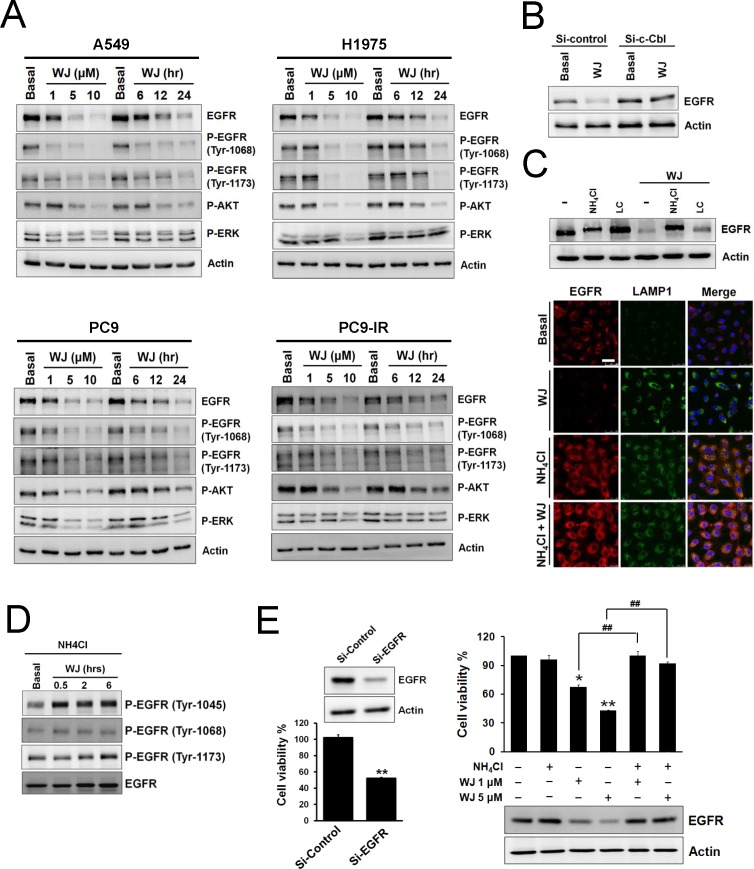
HDAC inhibitor reduced EGFR expression through c-Cbl-dependent lysosomal degradation pathway A, NSCLC cells were treated with 1, 5, 10 μM WJ for 24 hours, or treated with 5 μM WJ for 6, 12, 24 hours. Total cell lysates were prepared and western blot was performed using indicated antibiodies. B, Effect of siRNA-mediated knockdown of c-Cbl on WJ-induced EGFR degradation in A549 cells. C, A549 cells were pre-treated with 20 mM NH_4_Cl or 10 μM lactacystin (LC) for 30 minutes followed by 5 μM WJ for 24 hours (upper panel). EGFR associates with late endosome/lysosome (lower panel). A549 cells were treated with DMSO, 5 uM WJ, 20 mM NH_4_Cl or WJ plus NH_4_Cl for 24 hours. The evaluation of EGFR and LAMP1 expression were detected by laser scan confocal microscopy. Scale bars: 50 μm. D, Effect of phosphorylations of EGFR in A549 cells after WJ treatment. A549 cells were pre-treated with 20 mM NH_4_Cl for 30 minutes followed by 5 μM WJ for 0.5 to 6 hours. E, Effect of siRNA-mediated knockdown of EGFR on cell viability (left panel). A549 cells were transfected with EGFR siRNA or control (scramble) siRNA. ^**^P<0.01 versus control. Effect of NH_4_Cl on WJ-induced cytotoxicity (right panel). A549 cells were pre-treated with 20 mM NH_4_Cl for 30 minutes followed by WJ for 24 hours. The cell viability was measured by MTT assay. ^*^P<0.05, ^**^P<0.01 versus basal. ^##^*P*<0.01.

Since WJ could induce c-Cbl expression, its effect on EGFR was evaluated. WJ induced EGFR down-regulation in A549 cells, as well as SAHA and TSA did (Figure 2A and Supplementary Figure S1C). Its effect on wild-type or mutant EGFR lung adenocarcinoma cells was further evaluated. WJ down-regulated EGFR expression in all NSCLC cells and inhibited its downstream AKT and ERK in a dose- and time-dependent manner (Figure 2A). Knockdown of c-Cbl reversed WJ-mediated EGFR inhibition (Figure 2B), suggesting that WJ mediated EGFR inhibition through c-Cbl expression.

To investigate whether WJ-induced EGFR degradation was through c-Cbl-mediated lysosome pathway, lysosome inhibitor NH_4_Cl or proteasome inhibitor lactacystin was used. WJ-reduced EGFR expression was completely reversed by NH_4_Cl but partially reversed by lactacystin (Figure 2C), indicating the involvement of lysosome in this event. Confocal microscopy further demonstrated the induction of late endosome/lysosome marker LAMP1 by WJ, and the reverse of WJ-induced EGFR degradation by NH_4_Cl (Figure 2C). WJ was found to induce EGFR phosphorylation at Y1045, the docking site of c-Cbl, but not Y1068 or Y1173 (Figure 2D). These results indicated that c-Cbl mediated WJ-induced EGFR degradation through lysosome pathway. The effect of WJ on EGFR transcriptional regulation was also examined, and decreased EGFR mRNA by WJ was seen (Supplementary Figure S1D).

Knockdown of EGFR in A549 cells resulted in growth inhibition (Figure 2E). Lysosome inhibitor NH_4_Cl reversed WJ-induced EGFR degradation as well as growth inhibition (Figure 2E), indicating that EGFR plays an important role in HDAC inhibition-induced anti-cancer effect.

### WJ induced co-localization of c-Cbl and EGFR

**Figure 3:**
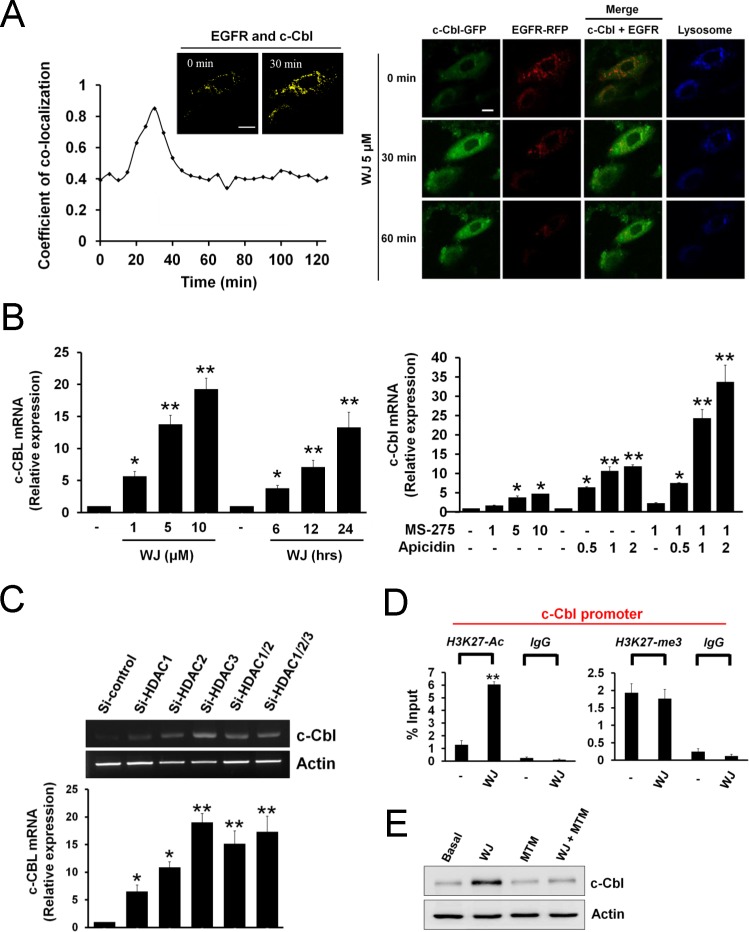
HDAC inhibition induced co-localization of c-Cbl and EGFR and promoted c-Cbl mRNA expression in lung adenocarcinoma cells A, A549 cells expressing c-Cbl-GFP (green) and EGFR-RFP (red) were exposed to 1 μM lysotraker (blue) for 30 minutes, and imaged for 120 minutes. Quantification of c-Cbl co-localized with EGFR (left panel). Plotted are the coefficient of co-localization between c-Cbl and EGFR at indicated times after treatment with 5 μM WJ. Co-localization between c-Cbl and EGFR in A549 cells after treatment with 5 μM WJ for 30 minutes. Co-localized signal is pseudo colored in yellow. Scale bars: 20 μm. Confocal microscopy of c-Cbl (green), EGFR (red) and lysosome (blue) in A549 cells after treatment with 5 μM WJ for indicated times (right panel). Scale bars: 20 μm. B, Induction of c-Cbl mRNA expression in A549 cells after WJ treatment (left panel). A549 cells were treated with WJ for indicated doses and time. Induction of c-Cbl RNA expression in A549 cells after MS-275 or apicidin treatment (right panel). The relative mRNA expression levels of c-Cbl were examined by Q-PCR. The levels of c-Cbl mRNA were normalized to level of GAPDH mRNA. ^*^P<0.05, ^**^P<0.01 versus basal. C, A549 cells were transfected with siRNA against HDAC1, HDAC2, HDAC3 or control (scramble) siRNA. ^*^P<0.05, ^**^P<0.01 versus basal. The mRNA expression level of c-Cbl was examined by RT-PCR (upper panel). The relative mRNA expression level of c-Cbl were also examined by Q-PCR (lower panel). The levels of c-Cbl mRNA were normalized to level of GAPDH mRNA. ^*^P<0.05, ^**^P<0.01 versus control. D, Histone acetylation and methylation within the c-Cbl promoter was regulated by WJ. Anti-H3K27-ac or anti-H3K27-me3 antibody was used in ChIP-qPCR to measure the levels of histone markers on c-Cbl promoter. Data were analyzed by the CT method and plotted as percent (%) input DNA. qChIP values were calculated by the following formula: percent (%) input recovery = 100 × 2 ^(input CT - bound CT)^. **P <0.01 versus basal. E, Effect of mithromycin A (MTM) on WJ-induced c-Cbl up-regulation. A549 cells were treated with 5 uM WJ, 1 uM MTM, or their combination for 24 hours. Total cell lysates were prepared and western blot was performed using indicated antibiodies.

Since WJ induced c-Cbl to degrade EGFR, the localization of c-Cbl and EGFR was evaluated in live cells by time-lapse confocal microscopy. The co-localization of c-Cbl and EGFR was observed at 30 minutes after WJ treatment (Figure 3A), and EGFR degradation ocurred at 60 minutes (Figure 3A and movie 1 in supplementary material), suggesting that WJ induced co-localization of c-Cbl and EGFR to degrade EGFR.

### Mechanism of WJ-induced c-Cbl expression

To elucidate WJ-induced c-Cbl up-regulation, c-Cbl mRNA was examined by Q-PCR, which showed increase in a dose- and time-dependent manner by WJ (Figure 3B). To investigate which HDAC isoform mediates c-Cbl expression, class I-specific HDACi MS-275 or apicidin was used and both induced c-Cbl mRNA expression (Figure 3B and Supplementary Figure S2A). Furthermore, knockdown of HDAC1, HDAC2 or HDAC3 as well as A549 HDAC6 −/− cells increased c-Cbl mRNA (Figure 3C and Supplementary Figure S2B), suggesting the involvement of HDAC1, 2, 3, and 6 in c-Cbl expression. Chromatin immunoprecipitation (ChIP) assay revealed that WJ induced acetylation but not methylation of histone H3 lysine 27 (H3K27) on the c-Cbl promoter (Figure 3D). There are putative SP1 binding sites within c-Cbl promoter by sequence analysis and SP1 inhibitor mythramycin A (MTM) was found to attenuate WJ-induced c-Cbl expression (Figure 3E), indicating the involvement of SP1 in c-Cbl transcription.

### WJ inhibited lung tumor growth *in vivo*

**Figure 4:**
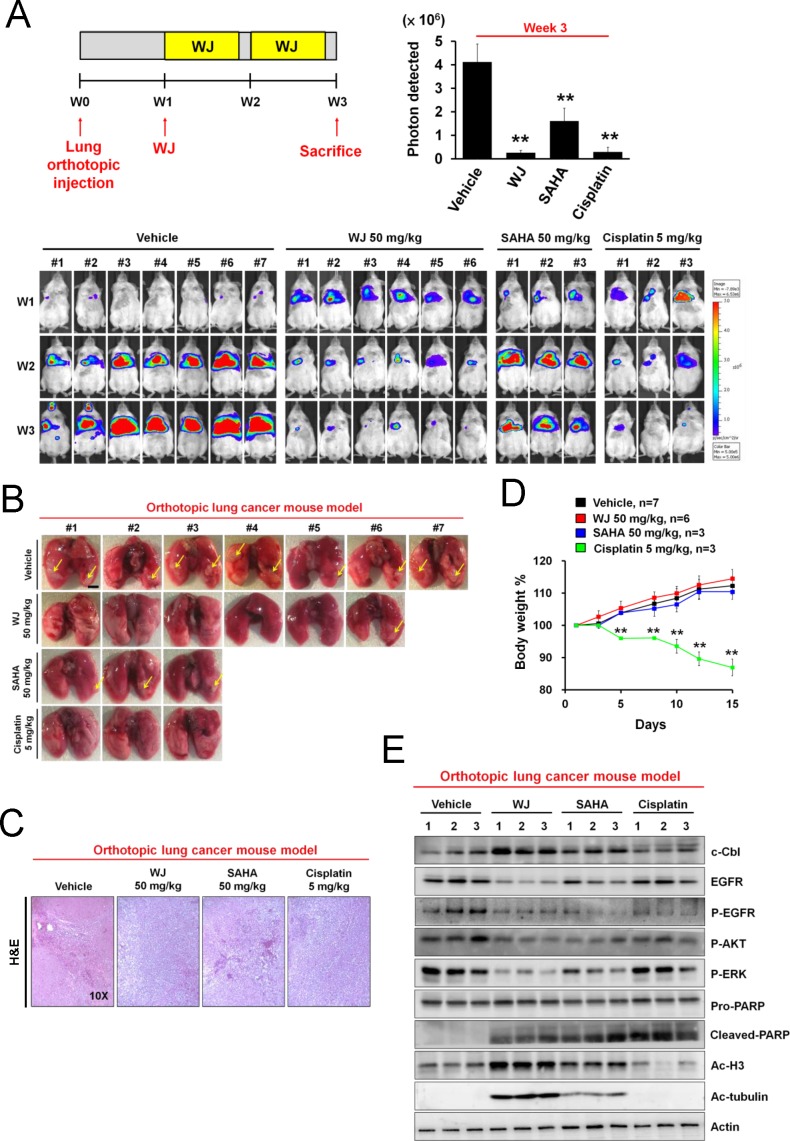
WJ inhibited lung tumor growth in an orthotopic lung adenocarninoma mouse model Male NOD/SCID mice were orthotopically injected with A549-Luciferase expressing cells (2 × 10^6^). WJ was injected at week 1 on days 1, 3 and 5 for two weeks. All mice were sacrificed at 3 weeks and lung segments were fixed by formalin. A, Schematic overview of WJ administration (upper left panel). The luciferase activity was detected with the noninvasive imaging system (IVIS imaging system, Xenogen) from week 1 to 3 (lower panel). Synchronized images were quantified at week 3 (upper right panel). **P<0.01 versus vehicle. B, Gross pictures of lungs. The arrowhead indicates the macroscopic lesions. Scale bars: 5 mm. C, Lung sections were counterstained with H&E. D, Changes in body weights. **P<0.01 versus vehicle. E, Effect of WJ, SAHA and cisplatin on PARP cleavage, EGFR signaling pathway and protein acetylation in lung tumor tissues. Total cell lysates were prepared and western blot was performed using indicated antibodies.

To evaluate the *in vivo* anti-tumor effect of WJ, A549-Luciferase expressing cells were orthotopically injected into the lung of NOD/SCID mice and photographed by IVIS imaging system at week 1, 2, and 3 (Figure 4A). Significant lung tumor growth was seen after three weeks injection. WJ and cisplatin inhibited tumor growth, which was confirmed by microscopic examination. Both agents were more effective than SAHA (Figure 4A-4C). However, cisplatin induced body weight loss (Figure 4D). Induction of c-Cbl, down-regulation of EGFR and inhibition of its downstream AKT and ERK accompanied with induction of PARP cleavage were seen in the lung tissues of WJ-treated group (Figure 4E). Acetylations of histone H3 and tubulin were also observed (Figure 4E).

**Figure 5:**
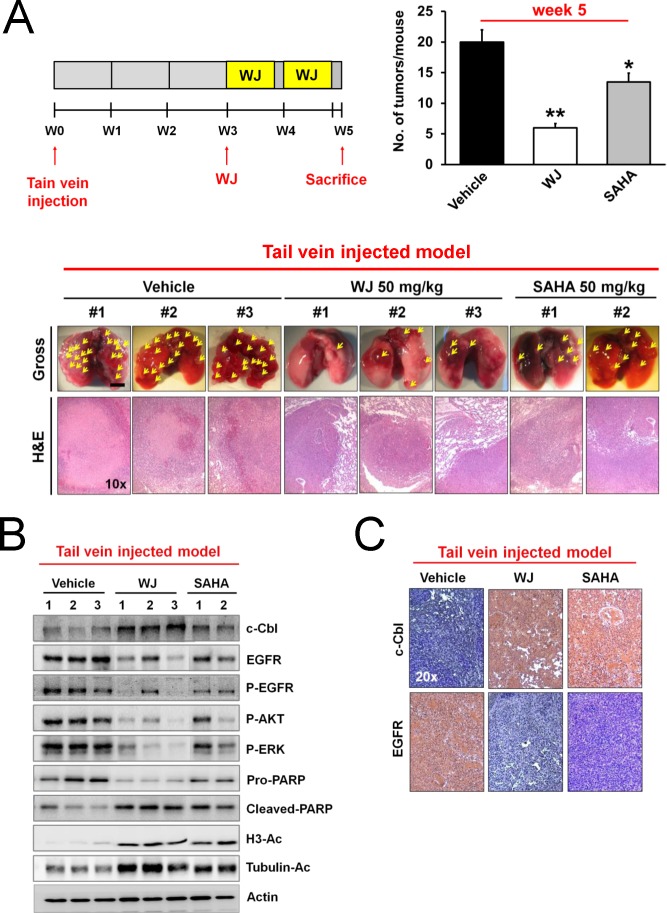
WJ inhibited lung tumor growth in a tail vein injected mouse model Male NOD/SCID mice were intravenously injected with CL1-5 cells (2.5 × 10^6^) through tail vein. WJ was intraperitoneally injected at day 21 on days 1, 3 and 5 for two weeks. All mice were sacrificed at 5 weeks and lung segments were fixed by formalin. A, Schematic overview of WJ administration (upper left panel). Numbers of tumor nodules per mouse (upper right panel). *P <0.05, **P <0.01 versus vehicle. Gross pictures and H&E stains of lungs (lower panel). The arrowhead indicates the macroscopic lesions (scale bar: 5 mm). B, Effect of WJ and SAHA on PARP cleavage, EGFR signaling pathway and protein acetylation in lung tumor tissues. Total cell lysates were prepared and western blot was performed using indicated antibodies. C, Lung tissues were immunostained with anti-c-Cbl or anti-EGFR antibody and representive results were shown.

To further examine the activity of WJ, CL1-5 cells were intravenously injected into NOD/SCID mice via the tail vein. Lung tumor nodules were evident three weeks after injection (Figure 5A). WJ was more potent than SAHA to inhibit tumor growth, which was confirmed by H&E staining (Figure 5A). WJ did not exhibit toxicity in various organs by microscopic evaluation (Supplementary Figure S3). Induction of c-Cbl accompanied with EGFR degradation in the lung tissues was evident by western blot and immunohistochemistry analysis (Figure 5B and 5C). WJ also reduced phosphorylations of EGFR and its downstream AKT and ERK accompanied with inducing PARP cleavage (Figure 5B). Acetylations of histone H3 and tubulin were confirmed (Figure 5B).

### Y1045-EGFR mutation abolished HDAC inhibitor-mediated anti-tumor effect in a orthotopic lung adenocarninoma mouse model

**Figure 6:**
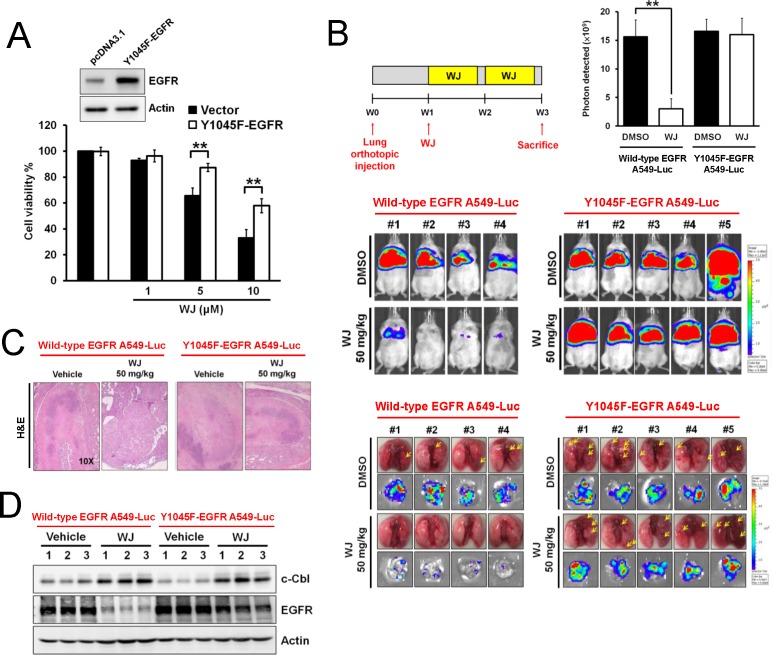
Y1045-EGFR mutation abolished HDAC inhibitor-mediated anti-tumor effect in an orthotopic lung adenocarninoma mouse model A, Effects of overexpressed Y1045F-EGFR mutation on WJ-mediated cell viability in A549 cells. Cells were treated with indicated doses of WJ for 48 hours. B, Schematic overview of WJ administration (upper left panel). Male NOD/SCID mice were orthotopically injected with A549-Luciferase expressing cells or Y1045F-EGFR-mutated cells (2 × 10^6^). WJ was injected at week 1 on days 1, 3 and 5 for two weeks. All mice were sacrificed at 3 weeks and lung segments were fixed by formalin. The luciferase activity was detected with the noninvasive imaging system (IVIS imaging system, Xenogen) from week 1 to 3 (middle panel). Synchronized images were quantified at week 3 (upper right panel). **P<0.01 versus vehicle. Gross pictures of lungs (lower panel). The arrowhead indicates the macroscopic lesions. Scale bars: 5 mm. Bioluminescence images of dissected spleens and livers from each mouse were shown. C, Lung sections were counterstained with H&E. D, Effect of WJ on c-Cbl and EGFR in lung tumor tissues. Total cell lysates were prepared and western blot was performed using indicated antibodies.

EGFR phosphorylation at Y1045 was the docking site for c-Cbl to induce EGFR degradation. To investigate the role of EGFR in HDAC inhibition-induced anti-cancer effect, Y1045F-mutant EGFR was overexpressed into A549 cells. WJ-induced growth inhibition was attenuated in Y1045F-mutant EGFR overexpressing cells (Figure 6A). The *in vivo* effect was examined by orthotopic lung injection of Y1045-mutant EGFR A549-Luciferase expressing cells into NOD/SCID mice (Figure 6B). The anti-tumor effect of WJ was abolished in Y1045F-mutant EGFR A549-Luc orthotopic lung tumors and WJ induced-EGFR degradation in wild-type EGFR A549 tumors was not seen in Y1045F-mutant EGFR A549 tumors (Figure 6B and 6C), indicating the critical role of EGFR in lung cancer formation. However, the induction of c-Cbl by WJ was not affected by Y1045F EGFR mutation (Figure 6D).

## DISCUSSION

We recently found that HDAC inhibitors (HDACi) act through transcription inhibition to induce EGFR down-regulation and growth inhibition in colon cancer cells [19]. In the present study, we further demonstrated that up-regulation of c-Cbl *in vitro and in vivo* mediated HDACi-induced EGFR degradation and anti-cancer effect on NSCLC. Knockdown of HDAC1, HDA2, HDAC3, or HDAC6 all induced c-Cbl up-regulation through mRNA transcription. SP1 is an important transcription factor closely associated cell growth and differentiation [20]. WJ-induced c-cbl up-regulation was reversed by SP1 inhibitor, suggesting the involvement of SP1 in c-Cbl expression. Our data showed loss of c-Cbl expression in lung cancer patients compared with their adjacent normal epithelium. Ectopic expression of c-Cbl induced growth inhibition and apoptosis as well as EGFR down-regulation in NSCLC cells, confirming the tumor suppressor role of c-Cbl. Therefore, c-Cbl up-regulation serving as a treatment against NSCLC contributes to anti-cancer effects of HDAC inhibitor in lung cancer. However, c-Cbl has been reported to be overexpressed in breast cancer and promote tumor progression by inhibiting tumor-suppressive TGF-β activity [21]. The role of c-Cbl on cancer formation might depend on different cellular context.

The great success of EGFR tyrosine kinase inhibitor (TKI) in NSCLC with EGFR activation mutation implies the critical role of EGFR in EGFR mutant NSCLC [22, 23]. The efficacy of EGFR TKI is modest in NSCLC without EGFR activating mutation [i.e. wild-type (wt) EGFR] and the role of EGFR in EGFR wt NSCLC is less addressed [24, 25]. We demonstrated that knockdown of EGFR inhibited NSCLC cell growth. Furthermore, mutation of EGFR at Y1045 attenuated WJ-induced cell growth inhibition as well as its anti-cancer effect and EGFR degradation *in vivo*, suggesting the important role of EGFR in EGFR wt NSCLC. Lysosome inhibitor NH_4_Cl also reversed both EGFR down-regulation and growth inhibition mediated by WJ. Therefore, HDACi mediates anti-NSCLC effect through c-Cbl-induced EGFR degradation. Our HDAC inhibitor is more potent than SAHA to exert *in vivo* anti-cancer effect and far less toxic than cisplatin, the backbone of chemotherapeutic agent against NSCLC. The expression of EGFR *per se* independent of kinase activity has been reported to stabilize the active glucose transporter, SGLT1, to prevent cell death [26]. By degradation of EGFR, WJ exerted promising anti-cancer effect like cisplatin. This effect might be resulted from EGFR degradation *per se* beyond TKI-induced EGFR inactivation. Therefore, HDAC inhibition-induced EGFR degradation might be a promising strategy for the treatment of EGFR wt NSCLC. A number of clinical trials regarding HDAC inhibitors for NSCLC are undergoing [16].

C-Cbl-mediated ubiquitination is the most elucidated pathway for EGFR degradation [1, 27, 28]. We found WJ induced c-Cbl up-regulation concomitant with EGFR down-regulation. Similarly, over-expression of c-Cbl induced EGFR down-regulation. However, c-Cbl knockdown reversed WJ-mediated EGFR degradation. Furthermore, WJ induced co-localization of c-Cbl and EGFR. Lysosome or proteasome was reported to be responsible for EGFR degradation [2, 28-30]. We also found lysosome inhibitor completely reverse WJ-induced EGFR degradation but partially done by proteasome inhibitor. All these suggested that WJ-induced EGFR degradation is c-Cbl-dependent and mainly through lysosome.

C-Met is a receptor tyrosine kinase that could be indirectly activated by EGFR to potentiate EGFR signalings in EGFR wt NSCLC cells [31]. Amplification of c-Met provides an escape mechanism of EGFR inhibition leading to TKI resistance in NSCLC [32]. Combined targeting c-Met and EGFR leads to an increase of xenograft antitumor activity in NSCLC [31]. Our data showed that WJ induced c-Met degradation in addition to EGFR down-regulation (Supplementary Figure S4A). Recently, the concept of synthetic lethality implied that combined inhibition of two or more genes leads to cell death whereas either one does not [33]. The exploration of synthetic lethality might be a approach to overcome drug resistance in cancer treatment [33]. Our data showed that WJ-induced c-Met degradation is c-Cbl-dependent and through endocytosis (Supplementary Figure S4B-S4D). However, lysosome or proteasome inhibitor both reversed this effect, suggesting the different destination of c-Cbl-mediated EGFR or c-Met degradation. The extent of protein ubiquitination might affect the route of degradation [34, 35]. For example, monoubiquitination facilitates receptor endocytosis and lysosomal degradation while polyubiquitination targets proteins for proteasomal degradation [34, 35]. It is possible that the extent of c-Cbl mediated ubiquitination of EGFR or c-Met determines the subsequent degradation route, which merits further investigation.

In addition to c-Cbl induction, several mechanisms are responsible for the inhibition of EGFR protein stability by HDACi. For example, LBH589 reduced EGFR expression in NSCLC cells through increasing the acetylation of heat shock protein 90 (HSP90), resulting in the dissociation of HSP90 and EGFR leading to EGFR degradation [36]. Inhibition of HDAC6 was found to enhance the endocytosis of EGFR through increasing tubulin acetylation [37, 38]. In this study, mRNA of EGFR and c-Met was also decreased by WJ (Supplementary Figure S4E), indicating that both decrease in EGFR protein stability and inhibition of EGFR transcription contribute to the anti-cancer effect of HDACi.

In conclusion, our findings showed that HDAC inhibitors act through *in vitro* and *in vivo* induction of c-Cbl to exert great activity against NSCLC. Therefore, Up-regulation of c-Cbl contributes to anti-cancer effects of HDAC inhibitor to serve as a treatment against NSCLC.

## MATERIAL AND METHODS

### Cell culture

A549 lung cancer cells, MEF, HS68 and Beas-2B were obtained from American Type Culture Collection (ATCC). CL1-0, CL1-5, CL83 and CL141 lung cancer cells were obtained from Dr. P.C. Yang (Graduated Institute of Clinical Medicine, National Taiwan University, Taipei, Taiwan). A549-Luc cells, which has previously been validated as stable clones, were obtained from Dr. P.W. Hsiao (Agricultural Biotech Research Center, Academia Sinica, Taipei, Taiwan). A549 and Beas-2B cells were cultured in Dulbecco's Modified Eagle's Medium. CL1-0, CL1-5, CL83, CL141, MEF and HS68 were cultured in RPMI 1640. A549-Luc cells were cultured in RPMI 1640 containing with 1μg/ml puromycin and 10% glutamate. All mediums were supplemented with 10% FBS. Cells were maintained at 37°C in 5% CO_2_.

### Chromatin immunoprecipitation (ChIP) assay

A549 cells were treated with 5 μM WJ for 24 hours, cross-linked with 1.42% formaldehyde for 15 minutes, collected by scraping in PBS and then centrifuged and lysed in 1 ml of IP buffer (150 mM NaCl, 50 mM Tris-HCl pH 7.5, 5 mM EDTA, 0.5% Nonidet P-40 and 1% Triton X-100) containing protease inhibitors. The obtained nuclear pellet was resuspended in IP buffer and sonicated. The sonicated lysates were immunoprecipitated with an H3K27-ac or an H3K27-me3 antibody, and the immune complexes were recovered using protein A-Sepharose (Roche). The immunoprecipitated DNA and input DNA were extracted by incubating the samples with 100 μl of 10% Chelex (Bio-Rad), then boiling them to reverse the cross-linking and centrifuging them to remove the Chelex slurry. For ChIP-on-chip assays, the DNA was further purified through phenol/chloroform/isoamyl alcohol extraction and ethanol precipitation. Real-time PCR was performed with the purified DNA using the following primers: 5′-GCCAACCTCCCGCCCACAAG-3′ and 5′-GTCTTCCCCGCCCCTA CGCC-3′.

### Transient transfection and siRNA knockdown

The plasmids and SmartPool siRNA (Dharmacon) were transfected with Lipifectamine 2000 according to the manufacturer's instructions. Briefly, 50% confluent cells in 6-well plate are transfected with indicated concentrations of plasmid or siRNA in 1 mL of serum-free medium for 6 h at 37°C. Then, 1 mL of medium containing 20% FBS is added to the transfection mixture. After 48 hours, cells are treated with WJ for 24 hours. The cells are lysed and the protein expression is analyzed by western blot.

### Immunofluorescent labeling and time-lapse confocal microscopy

To visualize the co-localization of EGFR and c-Cbl in live cells, A549 cells were transfected with GFP-c-Cbl, RFP-EGFR and labeling lysosome by lysotraker. Cells were viewed with a Zeiss LSM 780 confocal microscope. Live images were acquired every 15 minutes for 2 hours after treatment with WJ, and the results of Co-localized pixels were analyzed using LSM version software (Carl Zeiss).

### Development of tail-vein injected lung cancer models

Seven-week-old male NOD/SCID mice were obtained from the National Laboratory Animal Center. CL1-5 cells (2.5 × 10^6^) were injected intravenously into the tail vein of the mice. When lung tumor nodules were detected, the mice were treated intraperitoneally with WJ (50 mg/kg), SAHA (50 mg/kg) or vehicle (DMSO) 5 days a week for 2 weeks.

### Development of orthotopic lung cancer models

Seven-week-old male NOD/SCID mice were anesthetized by continuous flow of 2-3% isoflurane. A549-Luc cells (2 × 10^6^) suspended in 40 μl of PBS were injected percutaneously into the right lung of the mice using 500 μl Gas-Tight syringes with 27G top winged infusion set. These mice were then given endotoxon-free luciferse substrate and photographed using IVIS-200 imaging system once a week. When lung tumor nodules were detected, the mice were treated intraperitoneally with WJ (50 mg/kg), SAHA (50 mg/kg) or vehicle (DMSO) 5 days a week for 2 weeks.

### Immunohistochemistry (IHC) assay

Immunohistochemistry was performed with One-step Polymer-HRP Detection Kit (Biogenex) on sections from 10% paraffin-embedded samples according to the manufacturer's protocols. Pictures were acquired using TissueFAXS (TissueGnostics).

### Statistical analysis

The SPSS program (SPSS Inc.) was used for all statistical analysis. Statistical analysis was performed by Student's *t*-test. *p*<0.05 (*) and *p*<0.01 (**) were statistically significant. Data shown were representatives of at least three independent experiments. Quantitative data are presented as means ± standard error (SE).

## SUPPLEMENTARY MATERIALS AND METHODS, TABLES AND FIGURES




