# Supplementary material for: Induction of c-Cbl contributes to anti-cancer effects of HDAC inhibitor in lung cancer
Source: Oncotarget. 2015 Mar 8;6(14):12481–92. doi: 10.18632/oncotarget.3489 (PMC4494952; doi:10.18632/oncotarget.3489)
Supplement: Supplementary file 1 [file oncotarget-06-12481-s001.pdf]

Induction of c-Cbl contributes to anti-cancer effects of HDAC inhibitor in lung cancer

Supplementary Materials and Methods

Materials

SAHA was purchased from Merck and MS-275 and apicidin were obtained from Sigma. LysoTracker® was obtained and purchased from Invitrogen. The antibody specific for c-Cbl was obtained from BD Transduction Laboratories. The antibodies specific for EGFR and c-Met were obtained from Santa Cruz. The antibody specific for acetyl-histone H3 was obtained from Millipore. The antibody specific for acetyl-tubulin was obtained from Sigma. The antibody specific for PARP1/2 and caspase 3 were obtained from Santa Cruz. The antibodies specific for phospho-EGFR (Tyr1045), phospho-EGFR (Tyr1173), phospho-AKT (Ser473) and phospho-ERK1/2 (Thr202/Tyr204) were purchased from Cell Signaling. The antibody specific for β-actin antibody was purchased from Gene Tex.

Western blot analysis

Following treatment with test compounds, cells were lysed on ice. Total cell lysates were centrifuged at 13,000 rpm for 15 min at 4 ^{o}C, and then subjected to SDS–PAGE using adequate percentage polyacrylamide gels. Immunoblotting was performed using specific antibodies to evaluate the expression of different proteins.

Cell proliferation assay

Cells were seeded at 3000 cells/well in 96-well plates and maintained for 14-16 hours, then treated with test compounds. After 72 hours, cells were washed with PBS then added medium containing MTT reagent (Sigma) at a final concentration of 0.5 mg/ml for 4 hours. Then, the medium was replaced with 200 μl of DMSO for 30 minutes, and using an ELISA reader (570 nm) to get the absorbance density values. The 50% of inhibition concentration (IC_{50}) of death cells was calculated.

Flow cytometry

Cells were treated with DMSO or indicated doses of WJ for 24 hours, adherent and floating cells were collected and washed with PBS. Cells were suspended thoroughly using 100 μL Fixation/Permeabilization buffer purchased from eBioscience. Then, cells were suspended with 100μL staining buffer containing rabbit IgG isotype control (Alexa Fluor® 488 Conjugate) or c-Cbl rabbit antibody (Alexa Fluor® 488 Conjugate) and then incubated at room temperature for 30 mins in dark. In continuous, cells were subjected to BD FACS Calibur cytometer with CellQuest software.

Real-time Polymerase Chain Reaction (Q-PCR)

Total RNA was isolated using Trizol reagent. Reverse transcription reaction was performed by 2 μg of total RNA, which was reverse transcribed into cDNA using oligo-dT. Real time PCR was performed with cDNA samples using the ABI Prism 7900 Sequence Detection System (Applied Biosystems). Primers were as follows: EGFR (forward primer, 5'-TTCCTCCCAGTGCCTGAAT-3'; reverse primer, 5'-GGTTCAGAGGCTGATTGTGAT-3'); c-Met (forward primer, 5'-CAGATGTG TGGTCCTTTG-3'; reverse primer, 5'-ATTCGGGTTGTAGGAGT CT-3'); c-Cbl (forward primer, 5'-GTCAAGTCGTGGTGGCACC-3'; reverse primer, 5'-CCTG GCTACATGGCTTTTTTG-3'); GAPDH (forward primer, 5'-AGCCACATCG CTCAGACAC-3'; reverse primer, 5'-GCCCAATACGACCAAATCC-3'). The data were normalized by GAPDH.

Table S1: Characteristics of patients with lung adenocarcinoma with low c-Cbl expressions.

Characteristics	N=11
Age, y	
<60	3
>60	8
Gender	
Male	5
Female	6
Stage	
I/II	8
III/IV	3
c-Cbl expression	
High[a]	0
Low[b]	11

[a]High, representative lung adenocarcinoma with intense cytosolic EGFR immunoreactivity (+ , scores 2, 3).

[b]Low, representative lung adenocarcinoma showing negative expression and almost absent EGFR immunoreactivity (- , scores 0, 1).

Table S2: IC_{50} values of WJ and SAHA in various non-small cell lung cancer cells and normal cells.

Cell lines	WJ (μM)	SAHA (μM)
A549	8.16 ± 0.95	9.39 ± 1.68
CL1-0	1.15 ± 0.02	3.81 ± 0.64
CL1-5	1.85 ± 0.06	4.81 ± 1.02
CL83	2.17 ± 0.49	4.21 ± 0.65
CL141	2.12 ± 0.30	2.98 ± 0.92
H1975	1.72 ± 0.73	-
PC9	0.76 ± 0.35	-
PC9-IR	0.78 ± 0.49	-
MEF	6.67 ± 1.80	3.85 ± 0.64
HS68	10.30 ± 0.39	4.63 ± 0.74

Figure S1

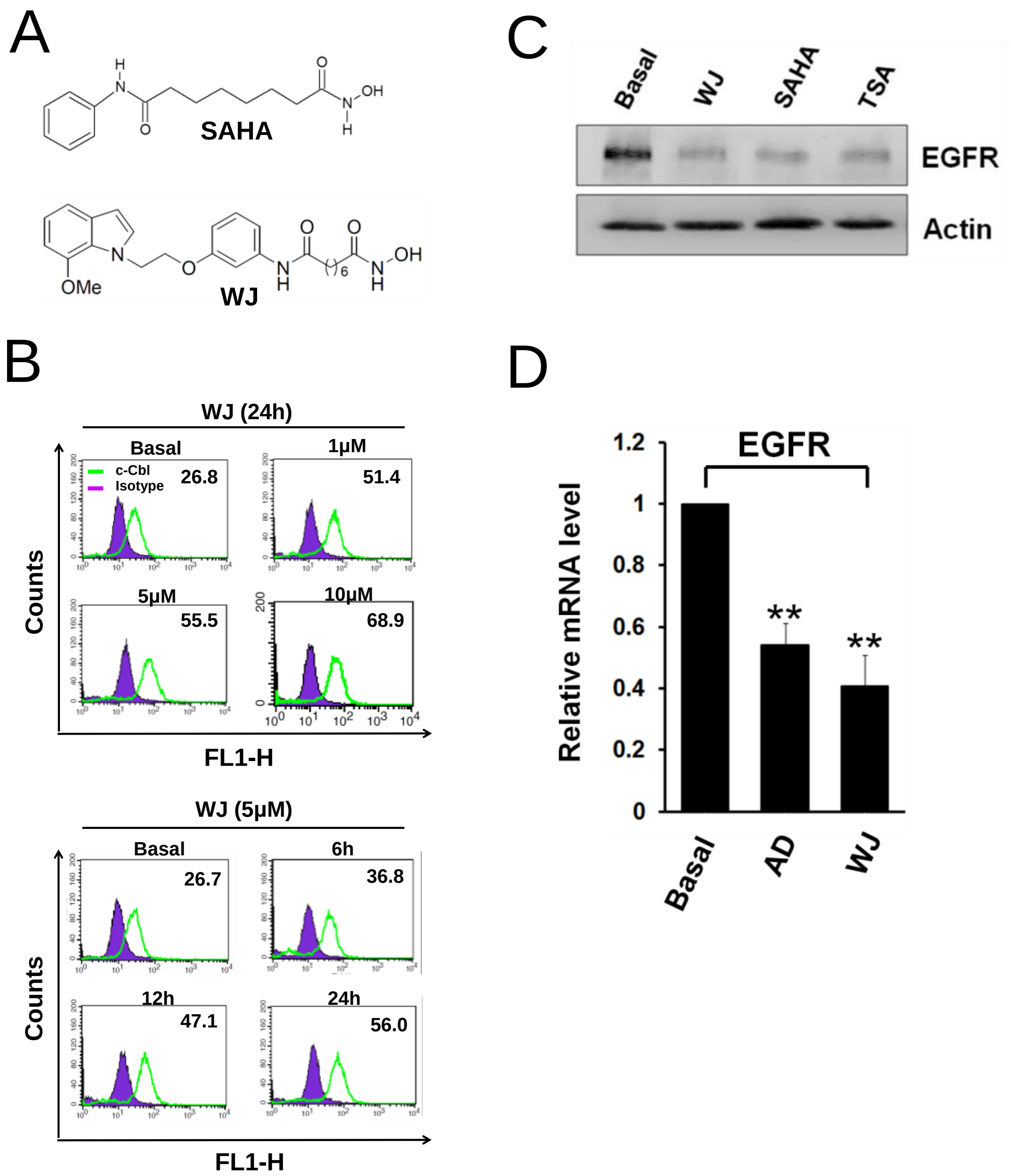

Figure S1: Effect of HDAC inhibitors on the expression of EGFR. A, Structure of WJ, SAHA and TSA. **B**, Up-regulation of c-Cbl by HDAC inhibition. A549 cells were treated with 1-10 µM WJ for 24 hours, or treated with 5 µM WJ for 6-24 hours. Cells were fixed and permealized then subjected to flow cytometry with indicated antibiodies. **C**, Effect of WJ, SAHA or TSA on EGFR expression in A549 cells. Cells

were treated with 5 μM WJ, 5 μM SAHA or 1 μM TSA for 24 hours. Total cell lysates were prepared and western blot was performed using indicated antibiodies. **D**, WJ decreased the mRNA level of EGFR. A549 cells were treated with 5 μM actinomycin D (AD) or 5 μM WJ for 24 hours, then mRNA was harvested and Q-PCR was performed. The levels of EGFR mRNA were normalized to level of GAPDH mRNA.

Figure S2

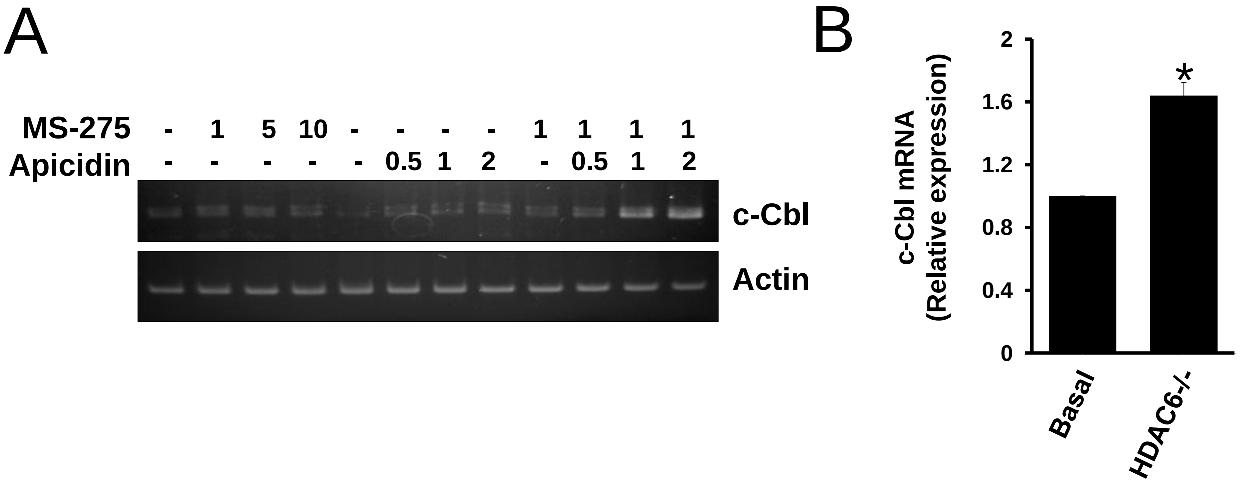

Figure S2: Class I and class II HDAC isoforms were involved in WJ-induced c-Cbl expression. A, Induction of c-Cbl RNA expression in A549 cells after MS-275 or apicidin treatment. A549 cells were treated with test compounds for indicated doses and time. The mRNA expression level of c-Cbl was examined by RT-PCR. **B**, The mRNA expression of c-Cbl between A549 wild-type and HDAC6-/- cells. $^{*}P<0.05$ versus basal.

Figure S3

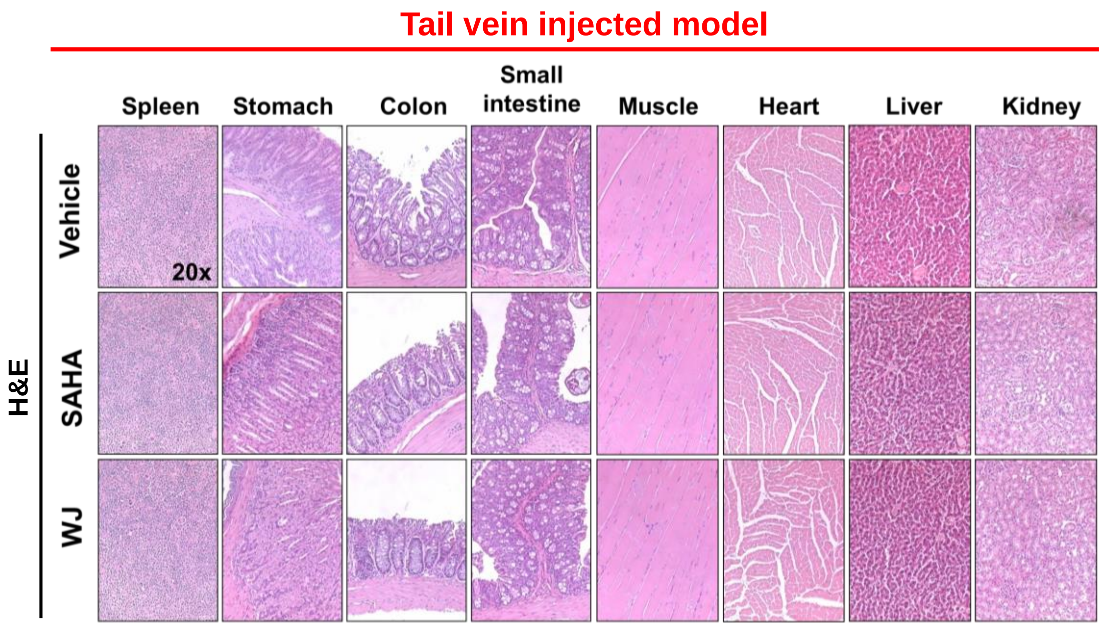

Figure S3: WJ did not exhibit toxicity in various organs by microscopic evaluation in a tail vein injected mouse model. Organ sections were counterstained with H&E.

Figure S4

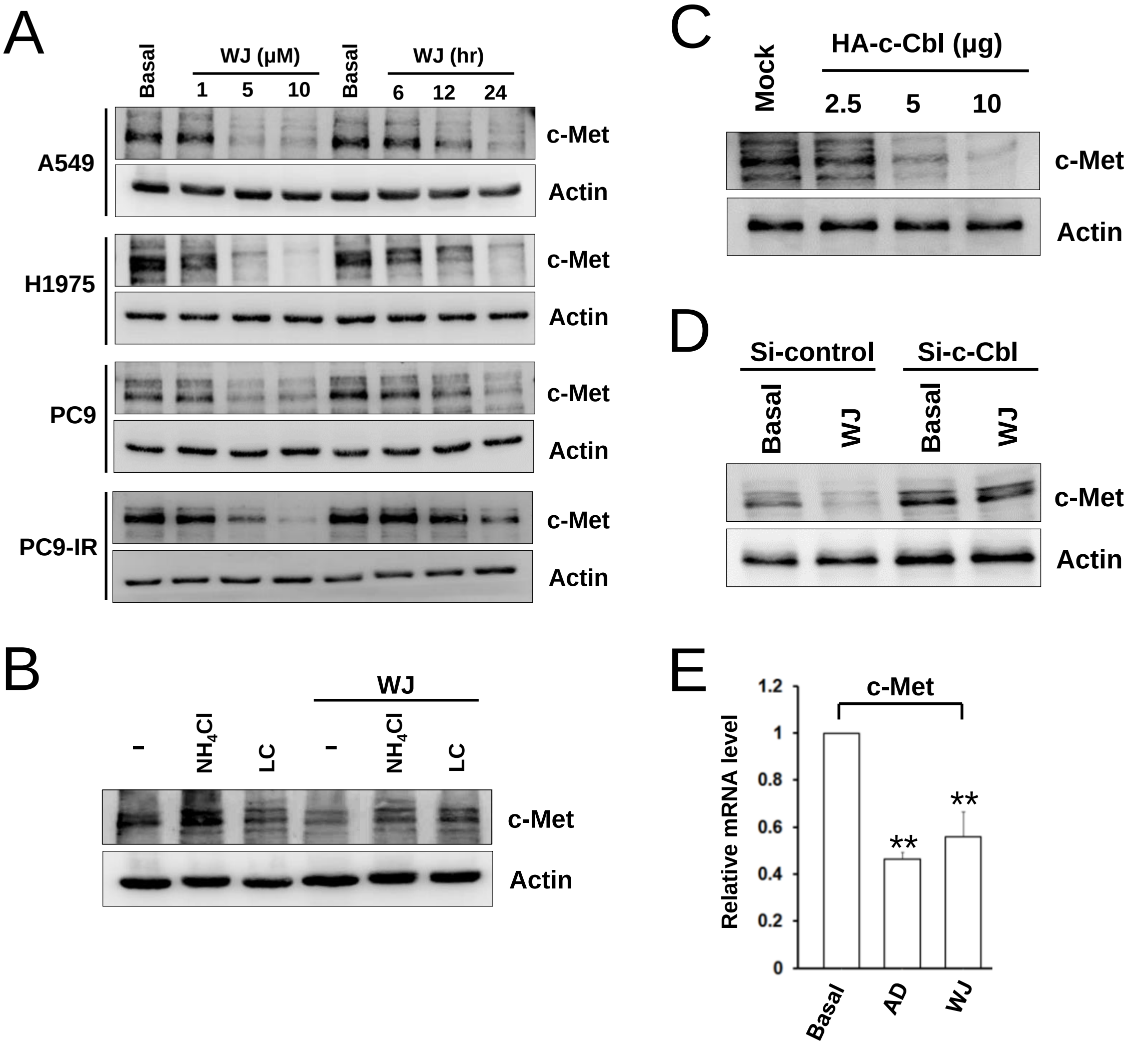

Figure S4: Effect of WJ on c-Met in various non-small cell lung cancer cell lines. **A**, WJ reduced c-Met expressions in various non-small cell lung cancer cells. Cells were treated with 1, 5, 10 μM WJ for 24 hours, or treated with 5 μM WJ for 6, 12, 24 hours. Total cell lysates were prepared and western blot was performed using indicated antibiodies. **B**, A549 cells were pre-treated with 20 mM NH_4Cl or 10 μM lactacystin for 30 minutes followed by 5 μM WJ for 24 hours. **C**, Dose-dependent effects of overexpressed c-Cbl on expressions of EGFR after 48 hours. **D**, Effect of siRNA-mediated knockdown of c-Cbl on WJ-induced degradation of c-Met. **E**, A549

cells were treated with 5μM actinomycin D (AD) or 5μM for 24 hours, then mRNA was harvested and Q-PCR was performed. The levels of c-Met mRNA were normalized to level of GAPDH mRNA. $^{**}P<0.01$ versus basal.
